# *Dcf1* Deficiency Attenuates the Role of Activated Microglia During Neuroinflammation

**DOI:** 10.3389/fnmol.2018.00256

**Published:** 2018-07-30

**Authors:** Jiao Wang, Jie Li, Qian Wang, Yanyan Kong, Fangfang Zhou, Qian Li, Weihao Li, Yangyang Sun, Yanli Wang, Yihui Guan, Minghong Wu, Tieqiao Wen

**Affiliations:** ^1^Laboratory of Molecular Neural Biology, School of Life Sciences, Shanghai University, Shanghai, China; ^2^Positron Emission Computed Tomography Center, Huashan Hospital, Fudan University, Shanghai, China; ^3^Institute of Nanochemistry and Nanobiology, Shanghai University, Shanghai, China; ^4^Shanghai Applied Radiation Institute, School of Environmental and Chemical Engineering, Shanghai University, Shanghai, China

**Keywords:** *Dcf1*, microglia, neuroinflammation, cytokines, migration, phagocytosis

## Abstract

Microglia serve as the principal immune cells and play crucial roles in the central nervous system, responding to neuroinflammation via migration and the execution of phagocytosis. Dendritic cell-derived factor 1 (Dcf1) is known to play an important role in neural stem cell differentiation, glioma apoptosis, dendritic spine formation, and Alzheimer’s disease (AD), nevertheless, the involvement of the *Dcf1* gene in the brain immune response has not yet been reported. In the present paper, the RNA-sequencing and function enrichment analysis suggested that the majority of the down-regulated genes in *Dcf1*^-/-^ (*Dcf1*-KO) mice are immune-related. *In vivo* experiments showed that *Dcf1* deletion produced profound effects on microglial function, increased the expression of microglial activation markers, such as ionized calcium binding adaptor molecule 1 (Iba1), Cluster of Differentiation 68 (CD68) and translocator protein (TSPO), as well as certain proinflammatory cytokines (Cxcl1, Ccl7, and IL17D), but decreased the migratory and phagocytic abilities of microglial cells, and reduced the expression levels of some other proinflammatory cytokines (Cox-2, IL-1β, IL-6, TNF-α, and Csf1) in the mouse hippocampus. Furthermore, *in vitro* experiments revealed that in the absence of lipopolysaccharide (LPS), the majority of microglia were ramified and existed in a resting state, with only approximately 10% of cells exhibiting an amoeboid-like morphology, indicative of an activated state. LPS treatment dramatically increased the ratio of activated to resting cells, and *Dcf1* downregulation further increased this ratio. These data indicated that *Dcf1* deletion mediates neuroinflammation and induces dysfunction of activated microglia, preventing migration and the execution of phagocytosis. These findings support further investigation into the biological mechanisms underlying microglia-related neuroinflammatory diseases, and the role of *Dcf1* in the immune response.

## Introduction

Neuroinflammation is widely regarded as a chronic innate immune response in the brain and a potentially pathogenic factor in a number of neurodegenerative diseases such as Alzheimer’s disease (AD), as well as traumatic brain injury (Panicker et al., 2015; Andreasson et al., 2016; Fernandez-Calle et al., 2017). Recently, due to its key signaling steps in the initiation of immune activation, greater attention has been paid to the potential of neuroinflammation as a therapeutic target (Panicker et al., 2015).

Microglia, which comprise approximately 20% of all glial cells, are the principal immune cells in the central nervous system and play a critical role in host defense against invading microorganisms and neoplastic cells (Rio-Hortega, 1932; Gonzalez-Scarano and Baltuch, 1999; von Bernhardi et al., 2015). In the normal adult brain, microglia display a remarkable branched, ramified morphological phenotype and are dispersed throughout the entire brain (Amor and Woodroofe, 2014). Upon injury, microglia undergo transformation to an amoeboid-like morphology, migrate to the site of injury, and execute phagocytosis (Andreasson et al., 2016). Microglia can also be activated by pathogen-associated molecules. Moreover, microglia also play a role in the regulation of activity-triggered synaptic plasticity and the remodeling of neural circuits, and further contribute to learning and memory (Koeglsperger et al., 2013; Parkhurst et al., 2013; Sofroniew, 2015). In the AD mouse brain, microglia have been shown to be clustered at the sites of Aβ plaques, with an activated, amoeboid-like morphology (Eisenberg and Jucker, 2012). Despite microglia being tightly packed and ubiquitously positioned in the tissue of young mice, coverage is impaired in old mice, and particularly more severely in 9-month-old APP_*Sw,Ind*_ Tg mice, leaving tissue devoid of microglial processes (Baron et al., 2014). It has been suggested that inflammation may be involved in the pathogenesis of AD (Miklossy, 2008). In the aged brain, microglia extend ramified processes into the surrounding tissue (Mosher and Wysscoray, 2014). A recent study using two-photon microscopy in the living brain of murine models of AD to examine microglial behavior, reported data showing that microglia in the aged brain were less motile and had fewer processes (Meyer-Luehmann et al., 2008), which supports the notion that aging is accompanied by impaired microglial function (Streit et al., 2008). However, despite recent progress, the understanding of the cellular and molecular mechanisms that mediate microglial activation is still far from comprehensive.

Dendritic cell-derived factor 1 (Dcf1) is a membrane protein that plays an important role in neural stem cell differentiation, glioma apoptosis, dendritic spine formation, and social interaction, as well as amyloid precursor protein metabolism (Wen et al., 2002; Wang et al., 2008; Ullrich et al., 2010; Xie et al., 2014; Liu et al., 2017a,b). Downregulation of the *Dcf1* gene facilitates differentiation of neural stem cells into astrocytes (Wang et al., 2008) and deletion of *Dcf1* leads to dendritic spine dysplasia in the mouse hippocampus (Liu et al., 2017a). Therefore, *Dcf1* is an important regulator of neural development.

It is known that certain neural development-regulating molecules also play important roles in the regulation of the immune response in the brain (Garay and McAllister, 2010). To explore the function of *Dcf1* in the neural immune system, we investigated the effect of *Dcf1* deletion on the activation of microglia and expression of proinflammatory cytokines under different conditions *in vitro* and *in vivo*. We found that *Dcf1* deletion produced profound effects on microglial function, increased the expression of microglial activation markers such as TSPO, Iba1, and CD68 as well as some proinflammatory cytokines, but decreased the migration and phagocytosis abilities of microglial cells and the expression levels of other proinflammatory cytokines.

## Materials and Methods

### Positron Emission Tomography (PET)

PET experiments were performed using a Siemens Inveon PET/CT system (Siemens Medical Solutions, Knoxville, United States) and conducted by the Huashan Hospital of China, according to the standard protocols and procedures (Kong et al., 2016). ^18^F-DPA-714 was given via the catheter system intravenously in a slow bolus. Isoflurane is an inhaled anesthetic that is mobilized through the respiratory tract and into the body of mice under the influence of oxygen. Dynamic PET was performed for 60 min on isoflurane-anesthetized male nude mice after intravenous injection of ^18^F-DPA-714. The experiments were carried out in compliance with national laws for the conduct of animal experimentation and were approved by the Animal Ethics Committee of Shanghai University.

### Immunohistochemical Staining

Brain samples from WT and *Dcf1*-KO mice (C57BL/6 male mice, 2–3 months-old) were cut by frozen sectioning. Slices were rinsed 3 times with PBS and permeabilized with 0.1% Triton X-100 in PBS for 40 min. The slices were subsequently blocked in 5% bovine serum albumin (Invitrogen, United States) in PBS at RT for 2 h, followed by incubation with a goat anti-Iba1 monoclonal primary antibody (1:500, Abcam, United States) at 4°C overnight. The following day, the slices were washed 3 times with PBS, incubated sequentially with a donkey anti-goat IgG secondary antibody Alexa 488 (1:1000, Abcam, United States) at RT for 2 h and the nuclear stain DAPI (Invitrogen, United States) at RT for 10 min, and finally washed 3 times with PBS. Fluorescence intensity was detected using a Zeiss LSM710 fluorescence microscope. All animals were treated in accordance with the guidelines of the Society for Neuroscience Ethics Committee on Animal Research. The study design was approved by the Animal Ethics Committee of Shanghai University.

### Cell Culture

BV2 cells, a mouse microglia cell line, were cultured in Dulbecco’s Modified Eagle Medium (Invitrogen, United States) supplemented with 10% fetal bovine serum (Invitrogen, United States) and 1% penicillin/streptomycin (Invitrogen, United States), and maintained at 37°C in a 95% humidified atmosphere with 5% CO_2_. At approximately 90% confluence, the cells were detached with 0.1% trypsin-EDTA (Invitrogen, United States), seeded onto appropriate plates with fresh medium, and incubated overnight.

### Transfection

BV2 cells were seeded onto 24-well plates at a density of 1 × 10^5^ cells/well and cultured overnight at 37°C in an atmosphere of 5% CO_2_. The following day, cells were transfected with the psiRNA-hH1neo plasmid or the psiRNA-*Dcf1* plasmid using *Lipofectamine*^TM^ 2000 (Invitrogen, United States), according to the manufacturer’s protocol.

### Observation of BV2 Microglia Cell Morphology

BV2 cells were cultured on 24-well plates and transfected as described above. 24 h post-transfection, cells were stimulated with 1000 ng/ml LPS for 12 h, followed by collection of bright-field images using a Nikon microscope. The cells were then rinsed with PBS, fixed in 4% paraformaldehyde in PBS at RT for 10 min, and permeabilized with 0.1% Triton X-100 in PBS for 10 min. The cells were subsequently blocked in 2% bovine serum albumin in PBS at RT for 1 h, followed by incubation with a goat anti-Iba1 monoclonal primary antibody (1:500, Abcam, United States) at 4°C overnight. The following day, the cells were washed 3 times with PBS, and incubated sequentially with a donkey anti-goat IgG secondary antibody Alexa 488 (1:1000, Abcam, United States) at RT for 2 h, the cytoskeleton red fluorescent probe ActinRed (1:50, KeyGEN BioTECH, China) at RT for 20 min, and DAPI at RT for 5 min, and finally washed 3 times with PBS. Fluorescence intensity was detected using a Zeiss LSM710 fluorescence microscope.

### Total RNA Extraction, cDNA Synthesis, and Real-Time Quantitative PCR (qPCR)

BV2 cells were cultured on 24-well plates and transfected as described above. 24 h post-transfection, cells were treated with 1000 ng/ml LPS for 12 h. Subsequently, the total RNA was extracted using a total RNA extraction kit (Promega, United States), according to the manufacturer’s protocol. The total RNA in the WT and *Dcf1*-KO hippocampal tissue was extracted in the same manner. The concentration of RNA was determined by measuring the absorbance at 260 nm, and 2 μg RNA was used for cDNA synthesis using an RT master mix (TaKaRa, Japan). QPCR amplification was performed in at least triplicate using a mixture of Top Green qPCR super mix (Transgen, China), cDNA samples, and designated primers (**Table 1**). The relative gene expression was calculated by comparing the CT value of the gene of interest with that of *Gapdh*, the internal control.

**Table 1:** List of primers used for qPCR.

Gene name	Primer sequence (5′–3′)
*Dcf1*	Upstream: CGCTGCTGCTGTTGACTATG
	Downstream: GTAGGTGTGCAAGGGGTAGG
*Ccl7*	Upstream: GCTGCTTTCAGCATCCAAGTG
	Downstream: CCAGGGACACCGACTACTG
*Cxcl1*	Upstream: CTGGGATTCACCTCAAGAACATC
	Downstream: CAGGGTCAAGGCAAGCCTC
*Csf1*	Upstream: ATGAGCAGGAGTATTGCCAAGG
	Downstream: TCCATTCCCAATCATGTGGCTA
*IL17D*	Upstream: AGCACACCCGTCTTCTCTC
	Downstream: GCTGGAGTTCGCACTGTCC
*Tnfsf11*	Upstream: CAGCATCGCTCTGTTCCTGTA
	Downstream: CTGCGTTTTCATGGAGTCTCA
*Cox*-2	Upstream: CAGTTTATGTTGTCTGTCCAGAGTTTC
	Downstream: CCAGCACTTCACCCATCAGTT
*IL-6*	Upstream: AACGATGATGCACTTGCAGA
	Downstream: CTCTGAAGGACTCTGGCTTTG
*IL-1β*	Upstream: CTTCCTTGTGCAAGTGTCTG
	Downstream: CAGGTCATTCTCATCACTGTC
*TNF-α*	Upstream: AAATTCGAGTGACAAGCCTGTAG
	Downstream: GAGAACCTGGGAGTAGACAAGGT
*Gapdh*	Upstream: TCACCACCATGGAGAAGGC
	Downstream: GCTAAGCAGTTGGTGGTGCA

### Western Blotting

The total protein in the WT and *Dcf1*-KO hippocampal tissue was extracted using cell lysis buffer (Beyotime, China), according to the manufacturer’s protocol. For protein extraction from BV2 cells, transfected cells cultured on 24-well plates were stimulated with 1000 ng/ml LPS for 12 h. Following the treatment, cells were washed twice with ice-cold PBS and the total protein was extracted using cell lysis buffer (Beyotime, China), according to the manufacturer’s protocol. Protein samples were separated by sodium dodecyl sulfate polyacrylamide gel electrophoresis and electroblotted onto nitrocellulose membranes. The membranes were blocked with 5% bovine serum albumin in PBS at RT for 1 h and then incubated with the following primary antibodies at 4°C overnight: goat anti-Iba1 (1:1000, Abcam, United States), rabbit anti-IL17D (1:500, Abcam, United States), rabbit anti-CD68 (1:500, Abways, China), rabbit anti-Cox-2 (1:500, Wanleibio, China), rabbit anti-IL-6 (1:500, Wanleibio, China), rabbit anti-IL-1β (1:500, Wanleibio, China), and rabbit anti-TNF-α (1:500, Wanleibio, China). The following day, the membranes were incubated with a mouse anti-GAPDH (1:1000, Abcam, United States) at RT for 1 h, followed by an infrared dye 700-conjugated goat anti-mouse IgG (1:10000, Zemed, United States) and either an infrared dye 800-conjugated goat anti-rabbit IgG (1:10000, Zemed, United States) or an infrared dye 700-conjugated donkey anti-goat IgG secondary antibody (1:10000, Zemed, United States) at RT for a further 1 h. Visualization and quantification was carried out using LI-COR Odyssey scanner and software (LI-COR Biosciences). The relative protein expression level was normalized to Gapdh of the same lane, and data were obtained from four independent immunoblots.

### Cell Migration Assay

BV2 cells were seeded at a density of 1 × 10^5^ cells per well on a 24-well plate and cultured for 24 h at 37°C with 5% CO_2_, followed by transfection as described above. 36 h post-transfection, a wound healing assay was used to evaluate alterations in the migration rate. Briefly, lineation was carried out at the central region of cell growth in each well using a P-20 pipette tip, and the cells were observed every 12 h for 48 h using a Nikon Ti-S fluorescence microscope. The results were analyzed using the Image Pro Plus software^1^.

### Cell Phagocytosis Assay

BV2 cells were seeded at a density of 1 × 10^5^ cells per well on a 24-well plate and cultured for 24 h at 37°C with 5% CO_2_, followed by transfection as described above. 36 h post-transfection, fresh culture medium containing 5 μl grapheme quantum dots (2–3 μm) was added to each well and incubated at 37°C for 5 min, followed by a PBS wash, fixation with 4% paraformaldehyde for 30 min and permeabilization with 0.1% Triton X-100 in PBS for 10 min at RT. Cells were subsequently incubated with ActinRed at a dilution of 1:50 for 30 min at RT and washed three times with PBS. The phagocytic activity of the cells was evaluated by confocal microscopy.

### Statistical Analysis

All data were analyzed using the Graphpad Prism software and were presented as the mean ± SEM. The mRNA and protein expression levels of WT and *Dcf1*-KO mice were analyzed using a *t*-test. The microscope images were analyzed using the Image Pro Plus software. The changes in cell morphology, mRNA and protein expression levels, and the migratory and phagocytic capacities of BV2 cells were analyzed using a one-way Analysis of Variance. Significance was set to *p* < 0.05.

## Results

### *Dcf1* Deletion Downregulates the Expression of Immune-Related Genes in the Hippocampus

**FIGURE 1:**
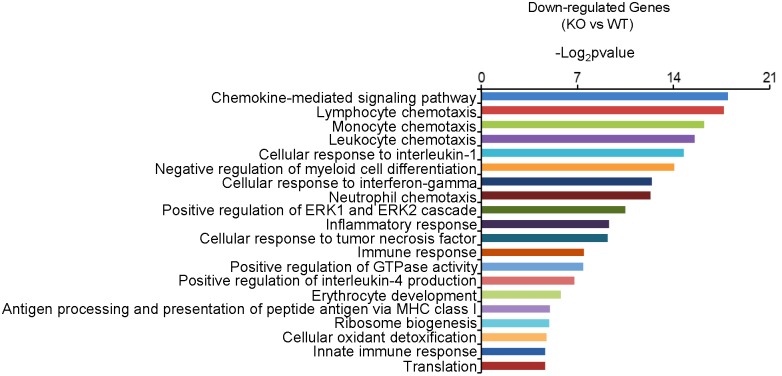
The function enrichment analysis of downregulated genes induced by *Dcf1* knockout using the DAVID platform. The gene function enrichment analysis of downregulated genes from RNA-sequencing results in WT and KO mice. The DAVID platform was used for analysis. The results showed that the majority of the downregulated genes were immune-related.

In order to gain insight into the molecular activities with which *Dcf1* may be involved in the nervous system, we examined and compared the mRNA levels in the hippocampus of both WT and *Dcf1*-KO mice by RNA sequencing and function enrichment analysis using DAVID (The Database for Annotation, Visualization, and Integrated Discovery). We found that the majority of downregulated genes in *Dcf1*-KO mice were immune-related (**Figure 1**). Since microglia are the major components of the immune system in the brain, we hypothesized that *Dcf1* may regulate microglial function. To test this hypothesis, we assessed the effects of *Dcf1* deletion on microglial activation and the production of cytokines in microglial cells using *Dcf1*-KO mice. In addition, the effects of *Dcf1* downregulation by RNAi on the LPS-induced changes in morphology, migratory and phagocytic capacity, and the expression levels of proinflammatory cytokines, in cultured BV2 cells were evaluated as described below.

### *Dcf1* Deletion Induces Microglial Activation *in Vivo*

**FIGURE 2:**
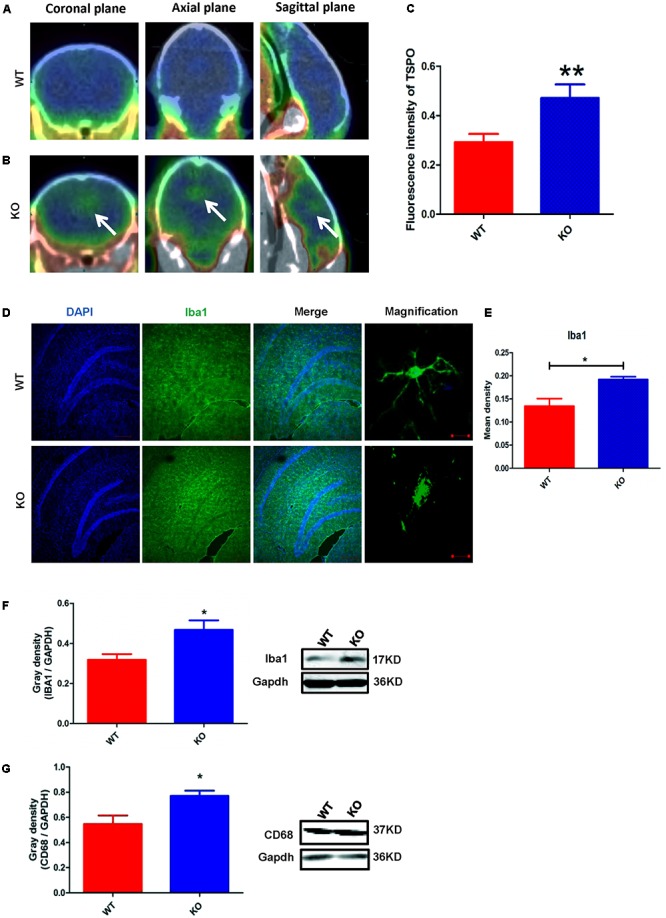
*Dcf1* deletion induces activation of microglial cells *in vivo*. ^18^F-DPA-714 (green) was used to trace TSPO (a biomarker of microglia) by PET to observe the activity of microglia *in vivo*. **(A,B)** Brain observation by PET in WT **(A)** and *Dcf1*-KO **(B)** mice. White arrowhead denotes activated microglia. **(C)** Quantitation of the TSPO in WT and *Dcf1*-KO mouse brain (Supplementary Table S1). **(D)** Immunohistochemical observation of microglia from WT and *Dcf1*-KO mouse brain sections. Microglial cells were detected by the Iba1 biomarker (green), and the nuclei were counterstained with DAPI (blue). Scale bars represent 200 μm. Higher magnification of confocal images were shown in right panel. Scale bars, 10 μm. **(E)** Quantitation of the mean density of Iba1 staining in WT and *Dcf1*-KO mouse brain sections (Supplementary Table S2) (mean ± SEM). *n* = 4. ^∗^*p* < 0.05. Protein expression of Iba1 **(F)** and CD68 **(G)** in WT and *Dcf1*-KO mouse brain tissue (Supplementary Tables S3, S4). Quantification of protein expression levels normalized to Gapdh. Data are expressed as the mean ± SEM. *n* = 3. ^∗^*p* < 0.05; ^∗∗^*p* < 0.01.

The activation of microglia is characterized by an increase in the expression level of TSPO (translocator protein) and Iba1 (ionized calcium binding adaptor molecule 1). Recently, a technique was developed to monitor the expression level of TSPO by PET imaging of a radiolabeled TSPO-binding tracer, ^18^F-DPA-714 (Auvity et al., 2017; Rizzo et al., 2017; Saba et al., 2017). Using this PET imaging technique, we found that TSPO expression was significantly increased in certain brain regions of *Dcf1*-KO mice, including the hippocampus, as compared with that of WT mice (**Figures 2A–C**), indicating that *Dcf1* deletion upregulated the ratio of activated microglia to resting microglia. Consistently, the expression level of Iba1 was also significantly increased in *Dcf1*-KO mice, as reflected by immunostaining and Western blotting analysis, which both show that the mean density in KO mice is 1.5 times that in WT mice (**Figures 2D–F**). Furthermore, Western blotting analysis shows that the expression level of CD68 was significantly increased in *Dcf1*-KO mice (**Figure 2G**).

### *Dcf1* Deletion Induces Abnormal Expression of Proinflammatory Cytokines *in Vivo*

**FIGURE 3:**
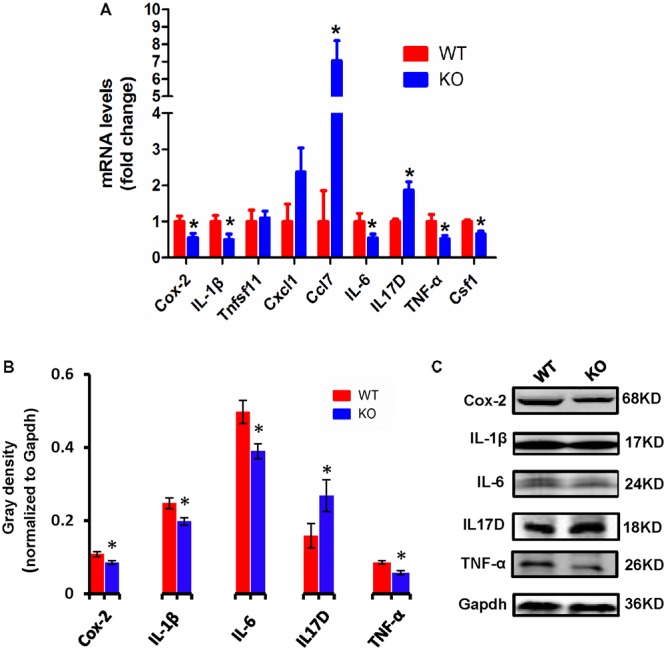
Expression of proinflammatory cytokines in WT and *Dcf1*-KO mice. **(A)** The mRNA levels of *Cox-2*, *IL-1β*, *Tnfsf11*, *Cxcl1*, *Ccl7*, *IL-6*, *IL17D*, *TNF-α*, and *Csf1* were assessed by qPCR in WT and KO mice. The relative abundance of each mRNA was expressed relative to *Gapdh*. Data are expressed as the mean ± SEM. *n* = 3. ^∗^*p* < 0.05 (Supplementary Table S5). **(B)** Protein expression of Cox-2, IL-1β, IL-6, IL17D, and TNF-α were assessed by Western blotting in WT and KO mice. Quantification of protein expression levels normalized to Gapdh. Datas are expressed as the mean ± SEM. *n* = 3. ^∗^*p* < 0.05 (Supplementary Table S6). **(C)** Sample Western blotting shown for *Cox-2*, *IL-1β*, *IL-6*, *IL17D*, *TNF-α*, *and Gapdh.*

Cytokines have been reported to promote neuronal differentiation and remodeling in the brain (Jeon and Kim, 2016). Many reports have shown that proinflammatory cytokines were dramatically increased in activated microglial cells (Hwang et al., 2014). In the central nervous system, central cytokines such as IL-6, TNF-α, and IL-1β are secreted from microglia, and are considered to be involved in neuronal development and neuroplasticity (Moynagh, 2005; Jeon and Kim, 2016). Thus, to investigate the molecular differences in proinflammatory cytokines between WT and *Dcf1*-KO mouse hippocampal tissue, we examined both the mRNA and protein expression levels of Cox-2, IL-1β, Tnfsf11, Cxcl1, Ccl7, IL-6, IL17D, TNF-α, and Csf1. As illustrated in **Figure 3A**, the mRNA levels of *Ccl7* and *IL17D* dramatically increased by 2-fold in KO as compared with WT mice, in addition to *Tnfsf11* and *Cxcl1*, although the latter two were not significantly increased. Moreover, the mRNA levels of *Cox-2*, *IL-1β*, *IL-6*, *TNF-α*, and *Csf1* were significantly decreased by approximately 50% in the *Dcf1*-KO mice. Western blotting was used to verify these changes. As can be seen in **Figures 3B,C**, the protein expression levels of Cox-2, IL-1β, IL-6, and TNF-α were consistently significantly reduced in the *Dcf1*-KO mice as compared with the WT mice, and IL17D was significantly increased by approximately 40%.

### Downregulation of *Dcf1* Alters the LPS-Induced Morphological Change in Cultured BV2 Microglial Cells

**FIGURE 4:**
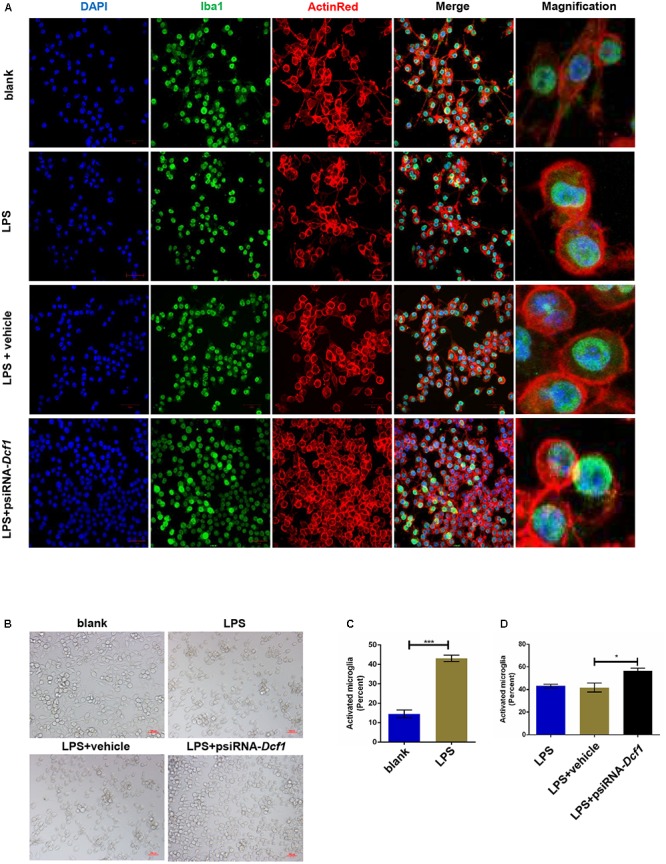
Morphology of LPS-stimulated BV2 microglial cells *in vitro*. BV_2_ microglial cells were transfected with the psiRNA-hH1neo plasmid or the psiRNA-*Dcf1* plasmid. 24 h post-transfection, BV2 microglia were stimulated with LPS (1000 ng/ml) and incubated for 12 h. **(A)** Immunofluorescence observation of the morphology in LPS-stimulated BV2 microglia *in vitro*. BV2 microglial cells were detected by the Iba1 marker (green), the cell skeleton by ActinRed (red), and the nuclei by DAPI (blue). Scale bars represent 50 μm. Higher magnification of confocal images were shown in right panel. Scale bars, 10 μm. **(B)** Bright field images of BV2 microglia. Scale bars represent 100 μm. **(C)** Comparison of the percentage of activated BV2 microglia stimulated with LPS (Supplementary Table S7). **(D)** Comparison of the percentage of LPS-activated BV2 microglia transfected with psiRNA-*Dcf1*. Data are expressed as the mean ± SEM. *n* = 6. ^∗^*p* < 0.05; ^∗∗∗^*p* < 0.001 (Supplementary Table S8).

To better understand the role that *Dcf1* plays in the changes in the microglial morphology induced by inflammatory stimulation, we investigated the effect of *Dcf1* downregulation on the morphological changes of cultured BV2 cells caused by LPS treatment. LPS is an outer membrane component of *Gram-negative* bacteria and a strong stimulator of microglial cells (Qin et al., 2004). As shown in Supplementary Figure S1 and Supplementary Table S13, psiRNA-*Dcf1* plasmid was used to knock down the *Dcf1* gene, and qPCR analysis of the *Dcf1* mRNA level revealed a significant decrease in the LPS + psiRNA-*Dcf1* group, with no significant differences seen among the other groups. Cell morphology was examined using immunostaining against ActinRed and Iba1. As shown in **Figure 4**, in the absence of LPS, the vast majority of cells were ramified and existed in a resting state, with approximately 10% of cells exhibiting an amoeboid-like morphology, indicative of the activated state. LPS treatment dramatically increased the ratio of activated to resting cells, with *Dcf1* downregulation further increasing this ratio. This result is consistent with the elevated activation of microglia cells seen in *Dcf1*-KO mice.

### Downregulation of *Dcf1* Affects the Expression of Cytokines *in Vitro*

**FIGURE 5:**
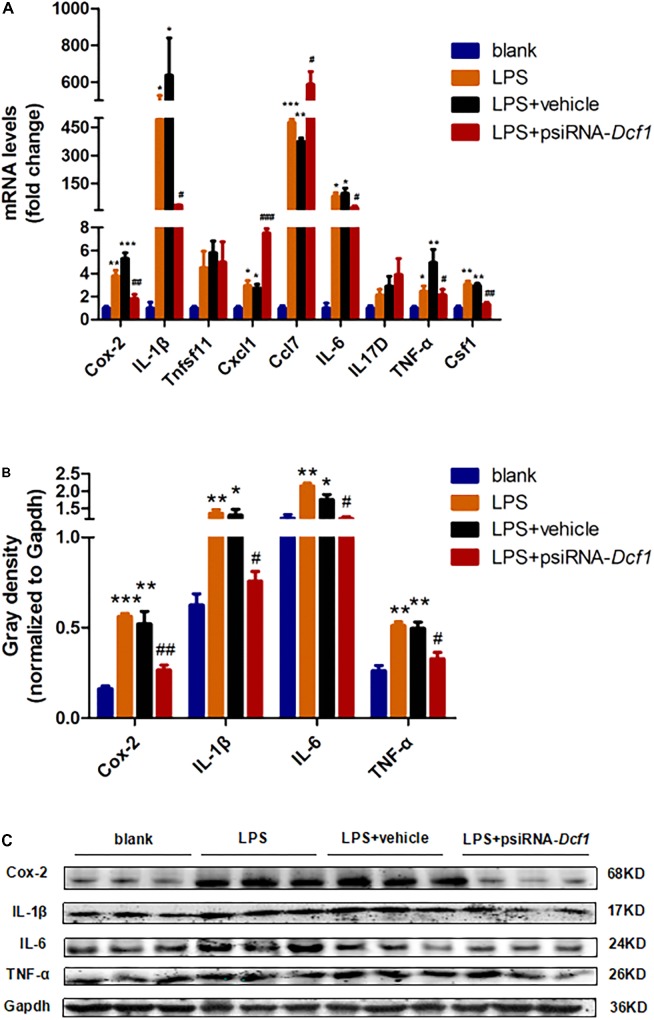
Effects of *Dcf1* downregulation on proinflammatory cytokines expression in LPS-stimulated BV2 microglia. BV_2_ microglial cells were transfected with the psiRNA-hH1neo plasmid or the psiRNA-*Dcf1* plasmid. 24 h post-transfection, BV2 microglia were stimulated with LPS (1000 ng/ml) and incubated for 12 h. **(A)** The expression of *Cox-2*, *IL-1β*, *Tnfsf11*, *Cxcl1*, *Ccl7*, *IL-6*, *IL17D*, *TNF-α*, and *Csf1* were assessed by qPCR. The relative abundance of each mRNA was expressed relative to *Gapdh* (Supplementary Table S9). **(B)** Protein expression of Cox-2, IL-1β, IL-6, and TNF-α were assessed by Western blotting. Quantification of protein expression levels normalized to Gapdh. Data are expressed as the mean ± SEM. *n* = 3. ^∗^*p* < 0.05; ^∗∗^*p* < 0.01; ^∗∗∗^*p* < 0.001 vs. blank. ^#^*p* < 0.05; ^##^*p* < 0.01; ^###^*p* < 0.001 vs. LPS + vehicle (Supplementary Table S10). **(C)** Sample Western blots shown for *Cox-2*, *IL-1β*, *IL-6*, *TNF-α*, *and Gapdh.*

To examine whether the silencing of *Dcf1* affected the inflammatory response at the molecular level, qPCR was performed in order to quantify the mRNA expression of the nine cytokines examined above *in vivo* studies. The relative abundance of each mRNA was expressed relative to *Gapdh*. As illustrated in **Figure 5A**, the mRNA levels of *Cox-2*, *IL-1β*, *Cxcl1*, *Ccl7*, *IL-6*, *TNF-α*, and *Csf1* were significantly increased in the LPS + vehicle group as compared with the group in the absence of LPS (blank). However, downregulation of *Dcf1* in the LPS + psiRNA-*Dcf1* group significantly decreased the levels of *Cox-2*, *IL-1β*, *IL-6*, *TNF-α*, and *Csf1*. In contrast, *Cxcl1* and *Ccl7* were dramatically increased, and IL17D was also elevated, although not significantly, as compared with the LPS + vehicle group. Western blotting was performed to further confirm whether downregulation of *Dcf1* affected the expression of these cytokines. As shown in **Figures 5B,C**, cells treated with LPS had increased expression of proinflammatory cytokines such as Cox-2, IL-1β, IL-6, and TNF-α, however, these four proinflammatory factors were significantly reduced upon *Dcf1* downregulation as compared with the LPS + vehicle group. These data demonstrated that LPS-stimulated BV2 microglial cells had increased the expression of proinflammatory cytokines, whereas downregulation of the *Dcf1* dramatically decreased the expression of most factors that were detected in LPS-stimulated BV2 cells, with the exception of Cxcl1, Ccl7, and IL17D. These results suggested that downregulation of *Dcf1* suppressed the expression of the majority of proinflammatory factors in activated BV2 microglial cells, supporting the previous results (**Figure 3**) showing differences in the expression of these genes between the hippocampal tissue from WT and *Dcf1*-KO mice.

### Downregulation of *Dcf1* Decreases the Migratory Ability of Microglia

**FIGURE 6:**
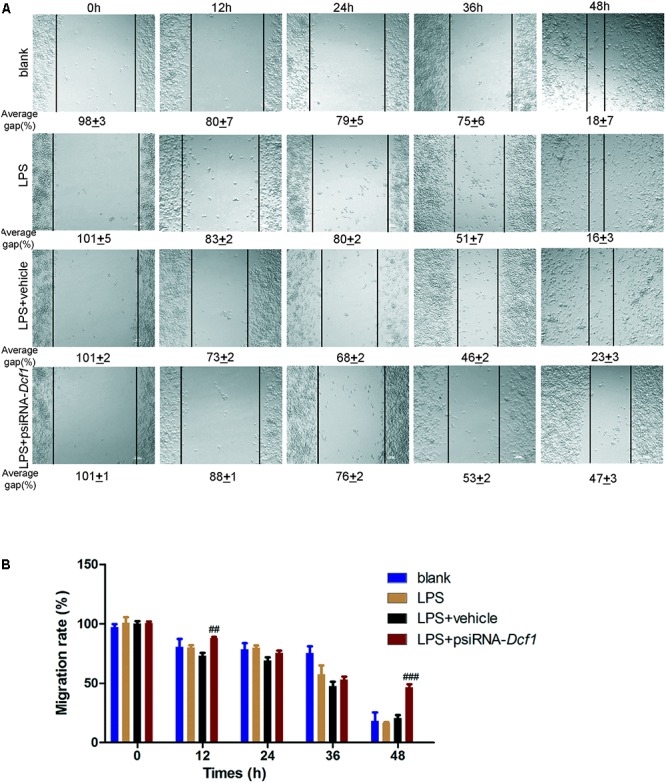
*Dcf1* deletion decreases the migratory capacity of BV2 microglial cells. BV2 microglial cells were transfected with the psiRNA-hH1neo plasmid or the psiRNA-*Dcf1* plasmid. 24 h post-transfection, BV2 microglia were stimulated with LPS (1000 ng/ml) and incubated for 12 h. **(A)** Representative images of the scratched areas in each condition at different time points were photographed. The average gap (AG, %) was used to quantify the relative migration of the cells. Scale bars represent 200 μm. **(B)** Statistical analysis of the BV2 microglial migration rate. Data are expressed as the mean ± SEM. *n* = 8. ^##^*p* < 0.01; ^###^*p* < 0.001 vs. LPS + vehicle (Supplementary Table S11).

Microglial cells respond to neuroinflammation with the processes of migration (Andreasson et al., 2016) and phagocytosis (Dheen et al., 2007). It has been shown that once activated, microglia migrate toward injured areas, and that this process is controlled by the presence of cytokines and chemokines (Noda and Suzumura, 2012). It can be speculated therefore, that *Dcf1* may affect microglial migration; and thus, a wound-healing assay was employed to study the effect of *Dcf1* downregulation on the migratory ability of BV2 cells. The representative images show the scratched areas of BV2 cells in different groups from 0 to 48 h (**Figure 6A**). Upon deletion of *Dcf1*, the migration rate of BV2 cells was increased by approximately 2-fold as compared with the LPS + vehicle group at 48 h. Statistical analysis reveals that *Dcf1* downregulation significantly decreased the migratory ability of BV2 cells (**Figure 6B**), suggesting that *Dcf1* may be involved in LPS-stimulated microglial cell migration.

### Downregulation of *D*cf1 in LPS-Activated Microglia Results in a Diminished Phagocytic Capacity

**FIGURE 7:**
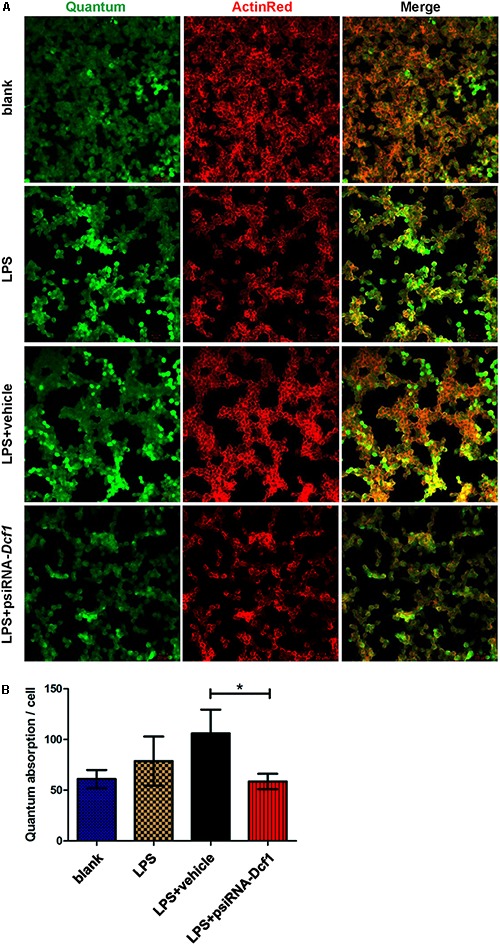
*Dcf1* deletion suppresses the phagocytic ability of BV2 microglia cells. BV2 microglial cells were transfected with the psiRNA-hH1neo plasmid or the psiRNA-*Dcf1* plasmid. 24 h post-transfection, BV2 microglia were stimulated with LPS (1000 ng/ml) and incubated for 12 h. **(A)** Image showing the phagocytic ability of BV2 microglia cells. Quanta were spontaneous green, and the cell skeleton was detected by ActinRed (red). Scale bars represent 20 μm. **(B)** Analysis of the average quantum absorption in each cell to assess the phagocytic activity of BV2 microglia. Data are expressed as the mean ± SEM. *n* = 4. ^∗^*p* < 0.05 (Supplementary Table S12).

To explore the effect of *Dcf1* downregulation on the phagocytic ability of LPS-activated microglia, a cell phagocytosis assay was performed. Following transfection, cells were incubated with medium containing green fluorescent grapheme quantum dots, and subjected to confocal microscopy. The cell skeleton was labeled with ActinRed (red) to form a composite image with which to count phagocytic cells (**Figure 7A**). The ratio of phagocytic cells to total cell number was calculated to evaluate the phagocytic capacity (**Figure 7B**). Image analysis shows that compared with the blank, the phagocytic capacity of BV2 cells following treatment with LPS was increased by approximately 10%. Moreover, downregulation of *Dcf1* led to a significant decrease of approximately 50% in microglial phagocytic ability as compared with the LPS + vehicle group.

## Discussion

**FIGURE 8:**
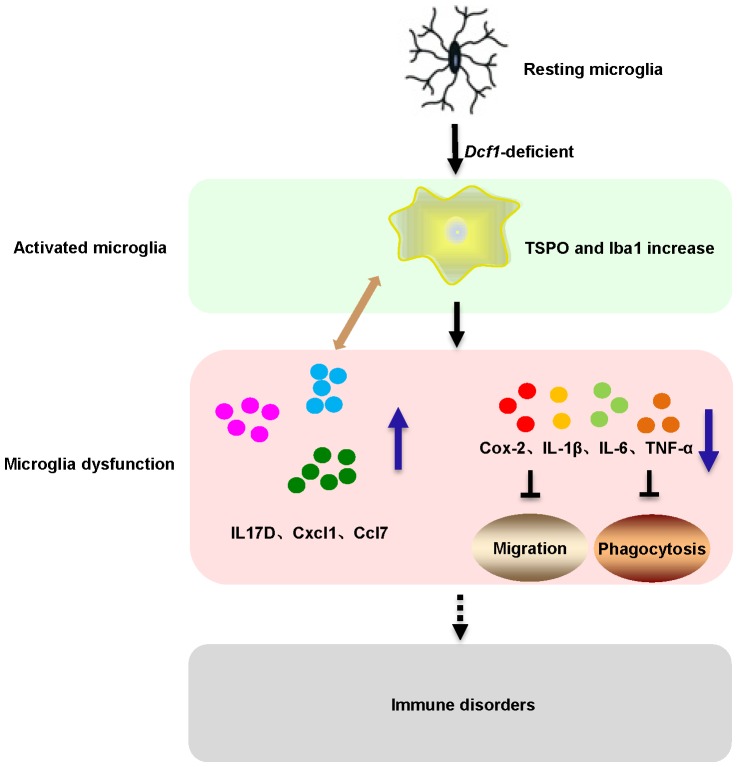
Schematic diagram of microglial activation and dysfunction induced by *Dcf1* deficiency. *Dcf1*-deficient activated microglia, induced subsequent aberrant proinflammatory cytokines release and microglial dysfunction, which blocked the migratory and phagocytic abilities of activated microglia.

Here, we provide evidence of neuroinflammatory responses induced by *Dcf1* deficiency (**Figure 8**). The RNA-sequencing and function enrichment analysis show that the majority of the downregulated genes in *Dcf1*-KO mice were immune-related (**Figure 1**), suggesting that *Dcf1* may play a role in brain immunity. Previous work has identified that microglial cells are responsible for surveillance immunity in the CNS and are activated in response to inflammation (Streit, 2002). TSPO is consistently raised in activated microglia of the CNS. In the present study, we found that *Dcf1* deficiency induced brain immunity and activation of microglial cells, as reflected by the upregulation of TSPO, IbaI, and CD68 *in vivo* (**Figure 2** and Supplementary Figure S2). *In vitro*, downregulation of *Dcf1* increased the morphological transformation of BV2 microglial cells to an amoeboid-like structure, which indicated an activated state (**Figure 4**). Following brain injury, microglial cells rapidly respond by activating the proinflammatory process, releasing inflammatory mediators and resolving the inflammatory response (Carniglia et al., 2017). The neuroinflammatory cytokines, Cxcl1, Ccl7, and IL17D were increased in *Dcf1*-KO mice (**Figure 3** and Supplementary Figure S3). Interestingly, a defect in *Dcf1* reduced the expression of other proinflammatory factors including Cox-2, IL-1β, IL-6, TNF-α, and Csf1 (**Figure 3**), implying that *Dcf1* influences multiple inflammatory responses. *In vitro*, downregulation of *Dcf1* decreased the expression of the majority detected cytokines, with the exception of Cxcl1, Ccl7, and IL17D (**Figure 5** and Supplementary Figure S4). Moreover, *Dcf1* knockdown diminished migratory (**Figure 6**) and phagocytic (**Figure 7**) abilities of BV2 cells, indicating that *Dcf1* deletion induced microglial dysfunction. Therefore, the deficiency of *Dcf1* induced an abnormal activation of microglial cells and disturbed the release of neuroinflammatory cytokines, which may destroy the immune homeostasis in the brain.

In order to assess the effects of *Dcf1* on microglia activation, PET technology was used to detect and monitor neuroinflammation *in vivo* (Auvity et al., 2017). TSPO was used as a biomarker for brain inflammation (Kong et al., 2016), since it is poorly expressed in the brain under normal physiological conditions, but is upregulated in activated microglial cells in response to inflammation or brain injury. Moreover, ^18^F-DPA-714, a novel TSPO radiotracer, has been used to detect and monitor neuroinflammation in various central system diseases (Wang et al., 2014; Lavisse et al., 2015; Kong et al., 2016). An increase in TSPO reflects increased microglial activation, which is a key event in the neuroinflammatory response (Dickens et al., 2014; Kong et al., 2016). **Figure 2** showed a PET image in which the green TSPO radiotracer was significantly increased and aggregated in *Dcf1*-KO mice, indicating activation of microglial cells. Moreover, morphological changes in mitochondria and the resulting dysfunction is one of the critical steps in neuroinflammation (Xie et al., 2014; Martins et al., 2015). It has been reported that TSPO is primarily located in the outer mitochondrial membrane, and our previous data have shown that *Dcf1* is also localized within mitochondria (Xie et al., 2014), suggesting that *Dcf1* may interact with TSPO in the mitochondria of microglial cells and influence their activation.

The neuroinflammatory cytokines Cxcl1, Ccl7, and IL17D were increased during local inflammatory reaction, which can expand the immune recruitment (Boissiere-Michot et al., 2014). Cxcl1 is a chemokine produced by glial cells that attracts immune cells to the brain (Wohleb et al., 2013; Miller and Raison, 2016), and Ccl7 has been reported to exert potent proinflammatory actions through chemotaxis of monocyte-derived macrophages and other inflammatory leukocytes in the central neural system (Stuart et al., 2015). The nature of these factors and their participation in neuroinflammatory responses (Johnson et al., 2011; Jung et al., 2015) are consistent with our results of increased *Cxcl1* and *Ccl7* (**Figure 3**) accompanied by the activation of microglia (**Figure 2**). This process also included an increase in IL17D (**Figure 3**), implying an important function of IL17D during microglial activation. Moreover, Cox-2, IL-1β, IL-6, TNF-α, and Csf1 are pleiotropic cytokines that are involved in various immune responses (Liew, 2003; Kishimoto, 2006; De et al., 2014; Leppkes et al., 2014), the levels of which were decreased in both *Dcf1*-KO mice (**Figure 3**) and LPS-stimulated BV2 cells (**Figure 5**). The same phenomena were also observed in BV2 microglial cells activated by Prostaglandin E2 (Petrova et al., 1999), where the mRNA levels of *IL-6* and *TNF-α* were modestly decreased. Reduced IL-1β did indeed inhibit the secretion of IL-6, TNF-α, and Cox-2; and high levels of IL-1β have been suggested to potentially induce the production of IL-6, TNF-α, and Cox-2 (Erta et al., 2012; Seibert and Masferrer, 1994). This is consistent with our results showing that lower expression levels of IL-1β may inhibit the secretion of IL-6, TNF-α, and Cox-2. The roles of central cytokines in the brain are not yet fully understood (Jeon and Kim, 2016), resulting in the paradox phenomena seen among different research studies (Felger and Lotrich, 2013; Farooq et al., 2017). Moreover, certain signaling pathways in dendritic cells, and immune cell types, have been shown to prevent the production of cytokines such as IL-6 (Hansen and Caspi, 2010), suggesting that *Dcf1* may also regulate the secretion of cytokines in the brain.

Microglia were activated *in vivo* detecting by ^18^F-DPA-714 in the *Dcf1*-KO brain (**Figure 2**), and silencing of *Dcf1* significantly promoted the LPS-induced changes in microglial morphology *in vitro* (**Figure 4**), suggesting that downregulation of *Dcf1* increased the activation of BV2 microglia cells. Iba1 is a microglia-specific protein (Ohsawa et al., 2004), and as such, in order to confirm the expression level of Iba1 in *Dcf1*-KO mice, immunohistochemical staining and Western blotting were performed. We found that the ratio of activated to resting microglial cells were dramatically increased, and the protein expression of Iba1 and CD68 was consistently elevated in *Dcf1*-KO mice (**Figures 2F,G**). It has previously been reported that the expression of Iba1 is regulated by cytokines and interferons (Imai and Kohsaka, 2002), and our data showed that the levels of the cytokines *Cxcl1*, *Ccl7*, and *IL17D*, were increased (**Figure 3A**), which may have induced a higher expression of Iba1. In addition, the levels of *Cox-2*, *IL-6*, *IL-1β*, *TNF-α*, and *Csf1* were all reduced (**Figure 3A**), implying that deletion of *Dcf1* interfered with the production of various inflammatory factors.

When neuronal damage occurs, microglia adopt an activated state and exert diversified functions including migration, phagocytosis, and the production of various cytokines and chemokines (Noda and Suzumura, 2012). We found that in the presence of LPS, downregulation of *Dcf1* decreased the migratory capacity of BV2 microglial cells compared with the LPS + vehicle group (**Figure 6**). Cell migration during the immune response appears to be modulated by two fundamental processes: cell adhesion systems located at the site of inflammation, and chemotactic signals elicited through cytokines and chemokines (Simson and Foster, 2000). It has also been reported that the release of inflammatory cytokines and chemokines initiates the inflammatory response and leads to the migration of microglia toward sites of injury (Zhou et al., 2014). In the present study, the decreased mRNA levels of the majority detected proinflammatory cytokines (**Figure 5**) in conjunction with the RNA sequencing results (**Figure 1**), in which the majority of downregulated genes were related to neuroimmune responses, showed a defect in the first step of microglial migration process. Therefore, the microglial migratory ability was reduced upon downregulation of *Dcf1* (**Figure 6**), which was accompanied by the downregulation of proinflammatory cytokines.

Our results showed that *Dcf1* deletion activated microglial cells in the brain *in vivo* (**Figures 2A,B**), and that downregulation of *Dcf1* led to a significant decrease in the phagocytic capacity of BV2 microglia *in vitro* (**Figure 7**). In contrast, enhanced microglia-activated phagocytosis has been proposed to be required for the removal of injured neurons, axons, and myelin sheaths (Fu et al., 2014). It has also been reported that cytokines was involved in the regulation of phagocytic capacity (Koenigsknecht-Talboo and Landreth, 2005; Tamura et al., 2017), and the activation of microglia induced cytokine secretion (such as IL-6 and TNF-α) and phagocytosis (Heo et al., 2015). However, our results showed that the downregulation of *Dcf1* inhibited the expression of the majority detected proinflammatory cytokines including IL-6 and TNF-α (**Figure 5**). Considering that the production of proinflammatory cytokines is typically accompanied by an increased phagocytic ability, it is reasonable to suggest that the decreased secretion of different proinflammatory cytokines impeded the phagocytic ability of microglia in the present study. Moreover, it has been reported that activated microglia migrate toward injured areas, and subsequently phagocytose foreign substances or unwanted self-debris (Noda and Suzumura, 2012). Furthermore, phagocytes such as microglia follow the cytokine gradient to the infected area (Strohmeyer et al., 2005). Our results indicated that the migratory ability of microglia was impaired by the downregulation of *Dcf1* (**Figure 6**), and that the diminished migratory ability maybe the first inhibitory step in the blockade of phagocytic capacity (**Figure 7**).

Interestingly, we were not the first to notice the paradox phenomena between activated microglia and the reduced secretion of proinflammatory cytokines, since it has been reported that deviations from microglial homeostasis induce diseases (Yirmiya et al., 2015). Our present results showed that *Dcf1* deletion decreased the expression of proinflammatory cytokines such as Cox-2, IL-1β, IL-6, Csf1and TNF-α (**Figure 3**), thus, we suggested that the downregulation of these proinflammatory cytokines (**Figure 3**) impaired the normal function of activated microglia, including the migratory (**Figure 6**) and phagocytic (**Figure 7**) abilities. Moreover, *Dcf1* deletion also increased the expression of other proinflammatory cytokines such as IL17D and Ccl7 (**Figure 3**), which may be secreted by the activated microglia. From these results, we can also speculate that the absence of *Dcf1* may induce the abnormal function of the immune system, causing an aberrant secretion of proinflammatory factors and block the normal immune response involving activated microglia. This demonstrates that the intimate details of abnormal inflammatory responses remain unclear, and that further research is required to determine the biological mechanisms induced by *Dcf1* deletion.

## Conclusion

In conclusion, our data indicates that *Dcf1*-deficient microglia induced aberrant proinflammatory cytokines release and subsequent microglial dysfunction, which blocked the migratory and phagocytic abilities of activated microglia. Taken together, these observations provide novel insight into the role of *Dcf1* in activated microglial cells during the neuroimmune response, and further lay the foundation for the elucidation of the mechanism underlying neuroinflammatory-related diseases.

## Author Contributions

JW, JL, and QW designed the experiments. JL, QW, and FZ conducted most of the experiments, with assistance from YK, QL, WL, YS, YW, and YG. JL, QW, and YK collected data and contributed to the statistical analysis. JW, JL, and QW analyzed the data and wrote the manuscript. JW, MW, and TW obtained funding and revised the manuscript. All authors read and approved the final manuscript.

## Conflict of Interest Statement

The authors declare that the research was conducted in the absence of any commercial or financial relationships that could be construed as a potential conflict of interest.

## Footnotes

1 http://dx.doi.org/10.17504/protocols.io.iascaee

## Abbreviations

*Ccl7*: chemokine (C-C motif) ligand 7
CD68: Cluster of Differentiation 68
*Cox-2*: cyclooxygenase-2
*Csf1*: colony stimulating factor 1
*Cxcl1*: chemokine (C-X-C motif) ligand 1
DAPI: 4′,6-diamidino-2-phenylindole
*Dcf1*: dendritic cell-derived factor 1
*Dcf1*-KO: *Dcf1*^-/-^
^18^F-DPA-714: N,N-Diethyl-2-(2-(4-(2-[^18^F]fluoroethoxy) phenyl)-5,7-dimethylpyrazolo [1,5-a]pyrimidin-3-yl) acetamide
Iba1: ionized calcium-binding adapter molecule 1
*IL-1β*: interleukin-1β
*IL-6*: interleukin-6
*IL17D*: interleukin 17D
LPS: lipopolysaccharide
PBS: phosphate-buffered saline
PET: positron emission tomography
qPCR: real-time quantitative PCR
RT: room temperature
*Tnfsf11*: tumor necrosis factor (ligand) superfamily, member 11
*TNF-α*: tumor necrosis factor alpha
TSPO: translocator protein
WT: wile-type
