# Supplementary material for: Dcf1 Deficiency Attenuates the Role of Activated Microglia During Neuroinflammation
Source: Front Mol Neurosci. 2018 Jul 30;11:256. doi: 10.3389/fnmol.2018.00256 (PMC6077288; doi:10.3389/fnmol.2018.00256)
Supplement: Supplementary file 1 [file Data_Sheet_1.pdf]

Dcf1 deficiency attenuates the role of activated microglia during neuroinflammation

Jiao Wang *, Jie Li, Qian Wang, Yanyan Kong, Fangfang Zhou, Qian Li, Weihao Li, Yangyang Sun, Yanli Wang, Yihui Guan, Minghong Wu , Tieqiao Wen

*Correspondence to:
Prof. Tieqiao Wen
wtq@shu.edu.cn.
Prof. Minghong Wu
mhwu@shu.edu.cn

Supplementary Figures:

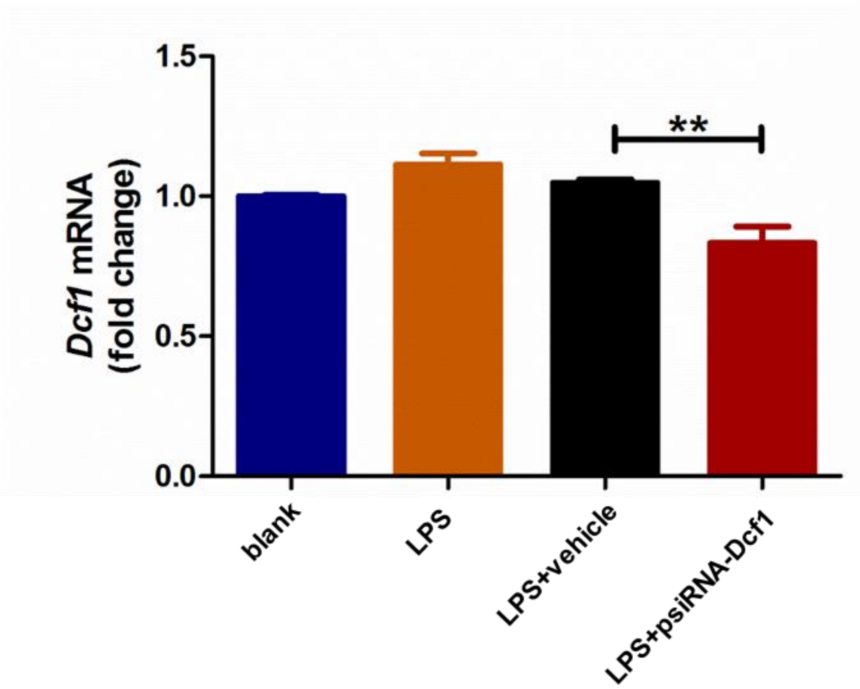

Supplementary Figure S1. _Dcf1_ knockdown efficiency in BV2 cells.

Plasmids were transfected 48 h prior to treatment with LPS for 12 h. *Dcf1* knockdown identification by qPCR. Abundance of the *Dcf1* mRNA transcript is expressed relative to *Gapdh* as an internal control. Data were expressed as the mean ± SEM. n= 4. *, p<0.05; **, p<0.01; ***, p<0.001 (**Supplementary Table 13**).

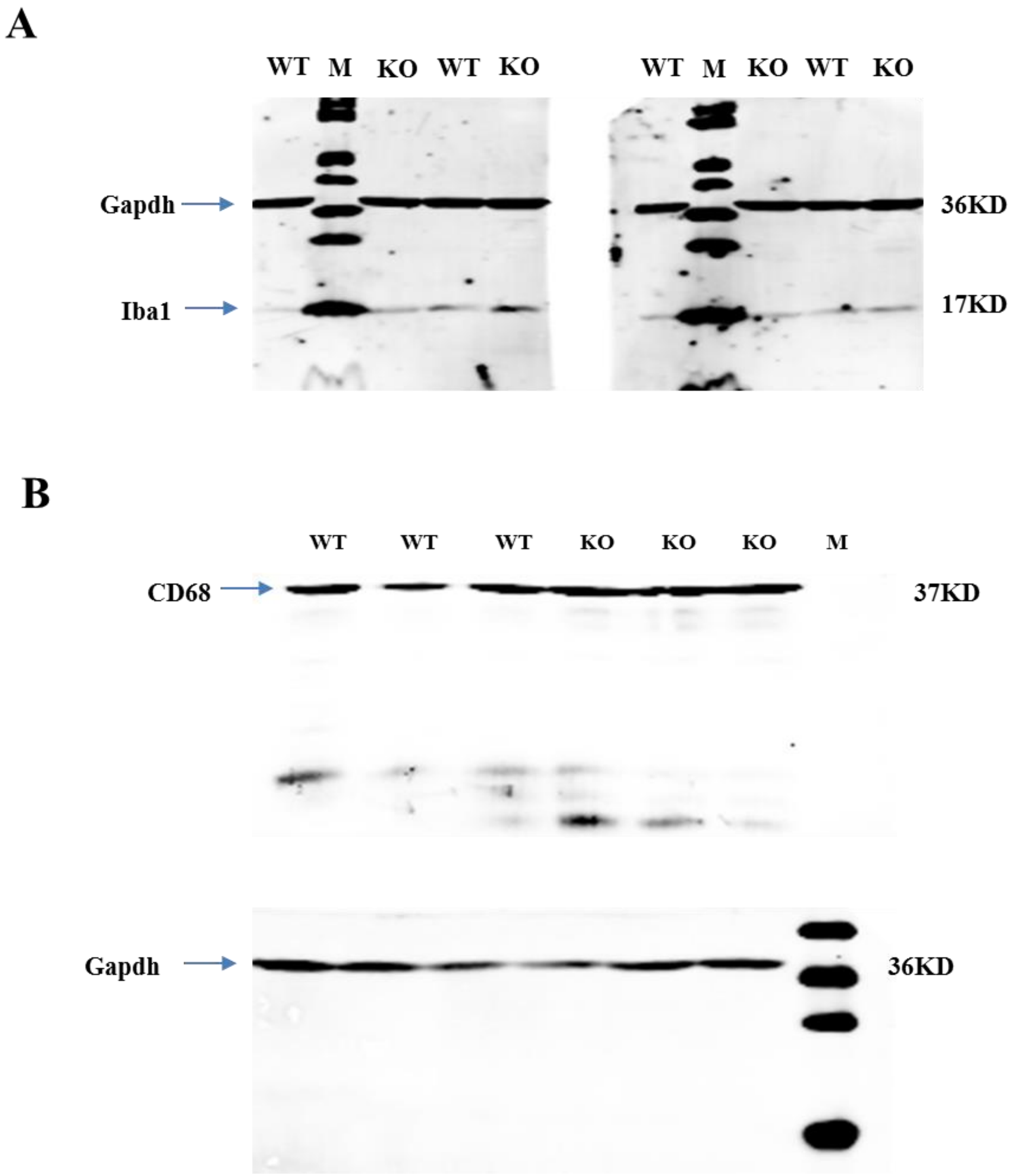

Supplementary Figure S2. ***Dcf1*** **deletion induces upregulation of microglial activation markers** ***in vivo***

Protein expression of microglial activation markers Iba1 **(F)** and CD68 **(G)** in WT and *Dcf1*-KO mouse brain tissue. Quantification of protein expression levels normalized to Gapdh.

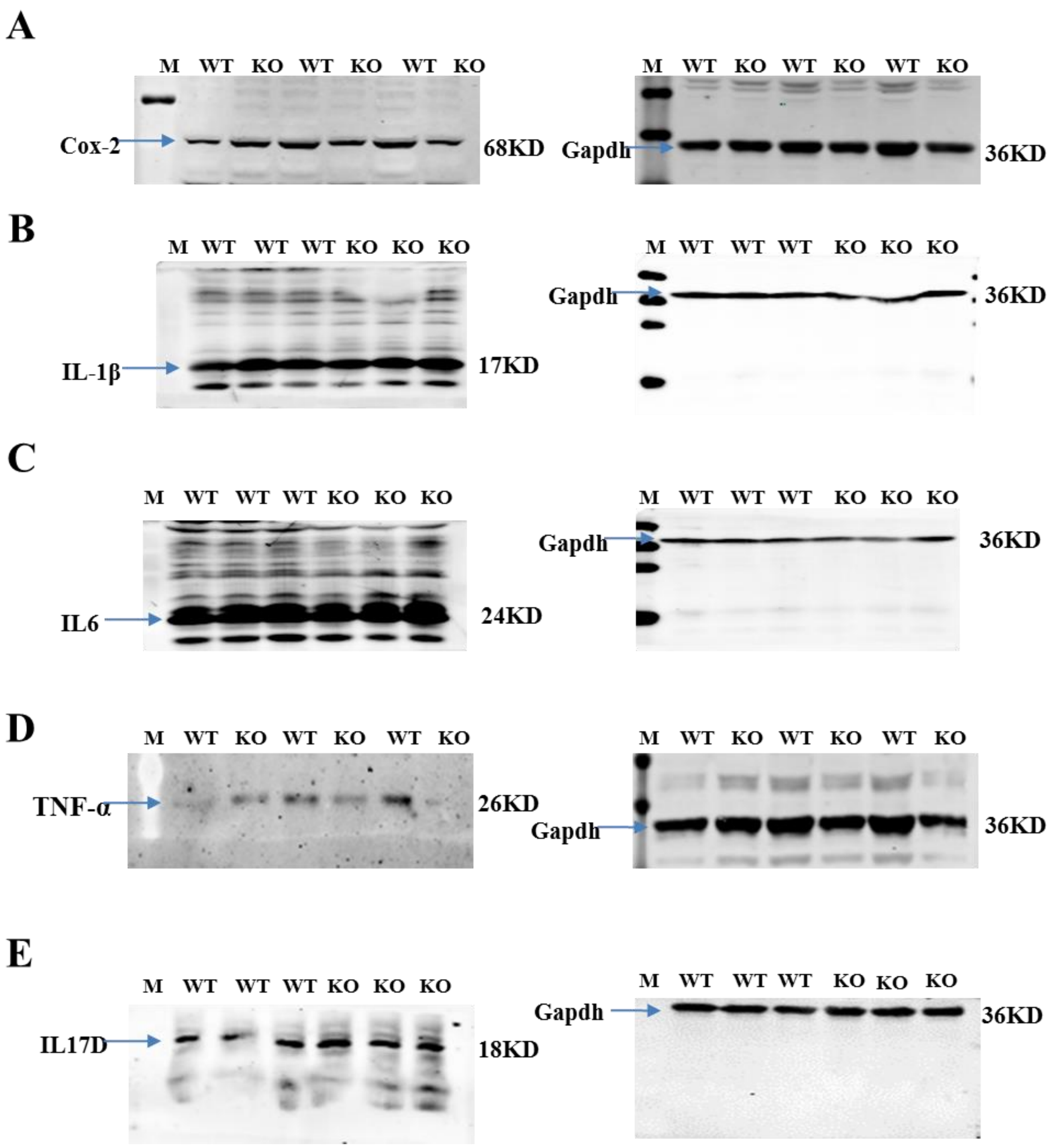

Supplementary Figure S3. Expression of proinflammatory cytokines in WT and *Dcf1*-KO mice

Protein expression of Cox-2**(A)**, IL-1β**(B)**, IL-6**(C)**, TNF-α**(D)** and IL17D**(E)**were assessed by Western blotting in WT and KO mice.

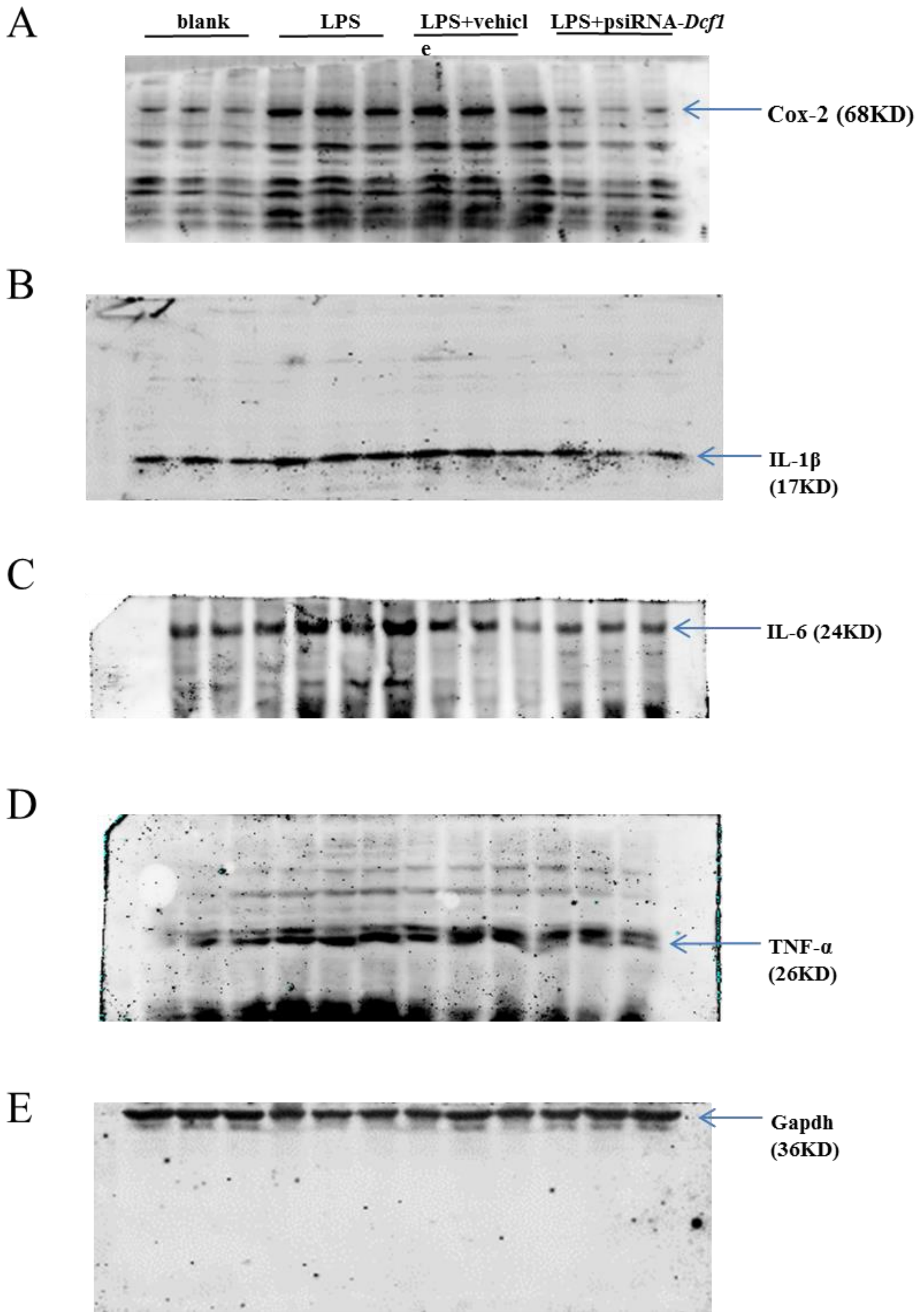

Supplementary Figure S4. Effects of *Dcf1* downregulation on proinflammatory cytokines expression in LPS-stimulated BV2 microglia

BV_2 microglial cells were transfected with the psiRNA-hH1neo plasmid or the psiRNA-*Dcf1* plasmid. 24 h post-transfection, BV2 microglia were stimulated with LPS (1000 ng/ml) and incubated for 12 h. Protein expression of COX-2**(A)**, IL-1β**(B)**, TNF-α **(C)** and IL-6**(D)** were assessed by Western blotting.

Table 1: t-test analysis of Figure. 2C

**Unpaired t-test**	**p value**	**Significant? p<0.05**	**Summary**
WT vs KO	0.0084	Yes	**

Data are expressed as the mean ± SEM. n = 3.

*, *p*<0.05; **, *p*<0.01; ***, *p*<0.001 vs. WT.

Table 2: t-test analysis of Figure. 2E

**Unpaired t-test**	**p value**	**Significant? p<0.05**	**Summary**
WT vs KO	0.0151	Yes	*

Data are expressed as the mean ± SEM. n = 4.

*, *p*<0.05; **, *p*<0.01; ***, *p*<0.001 vs. WT.

Table 3: t-test analysis of Figure. 2F

**Unpaired t-test**	**p value**	**Significant? p<0.05**	**Summary**
WT vs KO	0.0353	Yes	*

Data are expressed as the mean ± SEM. n = 3.

*, *p*<0.05; **, *p*<0.01; ***, *p*<0.001 vs. WT.

Table 4: t-test analysis of Figure. 2G

**Unpaired t-test**	**p value**	**Significant? p<0.05**	**Summary**
WT vs KO	0.0264	Yes	*

Data are expressed as the mean ± SEM. n = 3.

*, *p*<0.05; **, *p*<0.01; ***, *p*<0.001 vs. WT.

Table 5: t-test analysis of Figure. 3A

Unpaired t-test	p value	Significant? p<0.05	Summary
● *Cox2*:			
WT vs KO	0.0433	Yes	*
● *IL-1beta*:			
WT vs KO	0.0441	Yes	*
● *Tnfsf111*:			
WT vs KO	0.7862	no	ns
● *Cxcl1*:			
WT vs KO	0.2350	no	ns
● *Ccl7*:			
WT vs KO	0.0333	Yes	*
● *IL-6*:			
WT vs KO	0.0132	Yes	*
● *IL17D*:			
WT vs KO	0.0238	Yes	*
● *TNF-alpha*:			
WT vs KO	0.0364	Yes	*
● *Csf1*:			
WT vs KO	0.0200	Yes	*

Data are expressed as the mean ± SEM. n = 3.

*, *p*<0.05; **, *p*<0.01; ***, *p*<0.001 vs. WT.

Table 6: t-test analysis of Figure. 3B

Unpaired t-test	p value	Significant? p<0.05	Summary
● Cox2:			
WT vs KO	0.047751	Yes	*
● IL-1beta:			
WT vs KO	0.048958	Yes	*
● IL-6:			

WT vs KO	0.047189	Yes	*
● IL17D:			
WT vs KO	0.035709	Yes	*
● TNF-alpha:			
WT vs KO	0.012969	Yes	*

Data are expressed as the mean ± SEM. n = 3.

*, p<0.05; **, p<0.01; ***, p<0.001 vs. WT.

Table 7: t-test analysis of Figure. 4C

Unpaired t-test	p value	Significant? p<0.05	Summary
blank vs LPS	3.45x10^-5	Yes	***

Data are expressed as the mean ± SEM. n = 6.

*, p<0.05; **, p<0.01; ***, p<0.001 vs. blank.

Table 8: One-way ANOVA analysis of Figure. 4D

Bonferroni's Multiple Comparison Test	Significant? p<0.05	Summary
LPS vs LPS+vehicle	No	ns
LPS vs LPS+psiRNA-*Dcf1*	Yes	*
LPS+vehicle vs LPS+psiRNA-Dcf1	Yes	*

Data are expressed as the mean ± SEM. n = 6.

*, p<0.05; **, p<0.01; ***, p<0.001

Table 9: One-way ANOVA analysis of Figure. 5A

Bonferroni's Multiple Comparison Test	Significant? p<0.05	Summary
● *Cox2*:		

blank vs LPS	Yes	**
blank vs LPS+vehicle	Yes	***
blank vs LPS+psiRNA-*Dcf1*	No	ns
LPS vs LPS+vehicle	No	ns
LPS vs LPS+psiRNA-*Dcf1*	Yes	*
LPS+vehicle vs LPS+psiRNA-*Dcf1*	Yes	##
● *IL-1β* :		
blank vs LPS	Yes	*
blank vs LPS+vehicle	Yes	*
blank vs LPS+psiRNA-*Dcf1*	No	ns
LPS vs LPS+vehicle	No	ns
LPS vs LPS+psiRNA-*Dcf1*	No	ns
LPS+vehicle vs LPS+psiRNA-*Dcf1*	Yes	#
● *Tnfsf111* :		
blank vs LPS	No	ns
blank vs LPS+vehicle	No	ns
blank vs LPS+psiRNA-*Dcf1*	No	ns
LPS vs LPS+vehicle	No	ns
LPS vs LPS+psiRNA-*Dcf1*	No	ns
LPS+vehicle vs LPS+psiRNA-*Dcf1*	No	ns
● *Cxcl1* :		
blank vs LPS	Yes	*
blank vs LPS+vehicle	Yes	*
blank vs LPS+psiRNA-*Dcf1*	Yes	***
LPS vs LPS+vehicle	No	ns
LPS vs LPS+psiRNA-*Dcf1*	Yes	***
LPS+vehicle vs LPS+psiRNA-*Dcf1*	Yes	###
● *Ccl7* :		
blank vs LPS	Yes	***
blank vs LPS+vehicle	Yes	**
blank vs LPS+psiRNA-*Dcf1*	Yes	***
LPS vs LPS+vehicle	No	ns
LPS vs LPS+psiRNA-*Dcf1*	No	ns
LPS+vehicle vs LPS+psiRNA-*Dcf1*	Yes	#
● *IL-6*:		
blank vs LPS	Yes	*
blank vs LPS+vehicle	Yes	*
blank vs LPS+psiRNA-*Dcf1*	No	ns
LPS vs LPS+vehicle	No	ns

LPS vs LPS+psiRNA-*Dcf1*	No	ns
LPS+vehicle vs LPS+psiRNA-*Dcf1*	Yes	#
● *IL17D* :		
blank vs LPS	No	ns
blank vs LPS+vehicle	No	ns
blank vs LPS+psiRNA-*Dcf1*	No	ns
LPS vs LPS+vehicle	No	ns
LPS vs LPS+psiRNA-*Dcf1*	No	ns
LPS+vehicle vs LPS+psiRNA-*Dcf1*	No	ns
● *TNF-α* :		
blank vs LPS	Yes	*
blank vs LPS+vehicle	Yes	**
blank vs LPS+psiRNA-*Dcf1*	No	ns
LPS vs LPS+vehicle	No	ns
LPS vs LPS+psiRNA-*Dcf1*	No	ns
LPS+vehicle vs LPS+psiRNA-*Dcf1*	Yes	#
● *Csf1* :		
blank vs LPS	Yes	**
blank vs LPS+vehicle	Yes	**
blank vs LPS+psiRNA-*Dcf1*	No	ns
LPS vs LPS+vehicle	No	ns
LPS vs LPS+psiRNA-*Dcf1*	Yes	**
LPS+vehicle vs LPS+psiRNA-*Dcf1*	Yes	##

Data are expressed as the mean ± SEM. n = 4.
*, p<0.05; **, p<0.01; ***, p<0.001 vs.blank.
#, p<0.05; ##, p<0.01; ###, p<0.001 vs. LPS+vehicle.

Table 10: One-way ANOVA analysis of Figure. 5B

**Bonferroni's Multiple Comparison Test**	**Significant? p<0.05**	**Summary**
● Cox2:		
blank vs LPS	Yes	***
blank vs LPS+vehicle	Yes	**
blank vs LPS+psiRNA-*Dcf1*	No	ns
LPS vs LPS+vehicle	No	ns
LPS vs LPS+psiRNA-*Dcf1*	Yes	**
LPS+vehicle vs LPS+psiRNA-*Dcf1*	Yes	##

● IL-1β :		
blank vs LPS	Yes	**
blank vs LPS+vehicle	Yes	*
blank vs LPS+psiRNA-*Dcf1*	No	ns
LPS vs LPS+vehicle	No	ns
LPS vs LPS+psiRNA-*Dcf1*	Yes	*
LPS+vehicle vs LPS+psiRNA-*Dcf1*	Yes	#
● IL6:		
blank vs LPS	Yes	**
blank vs LPS+vehicle	Yes	*
blank vs LPS+psiRNA-*Dcf1*	No	ns
LPS vs LPS+vehicle	No	ns
LPS vs LPS+psiRNA-*Dcf1*	Yes	**
LPS+vehicle vs LPS+psiRNA-*Dcf1*	Yes	#
● TNF-α :		
blank vs LPS	Yes	**
blank vs LPS+vehicle	Yes	**
blank vs LPS+psiRNA-*Dcf1*	No	ns
LPS vs LPS+vehicle	No	ns
LPS vs LPS+psiRNA-*Dcf1*	Yes	*
LPS+vehicle vs LPS+psiRNA-*Dcf1*	Yes	#

Data are expressed as the mean ± SEM. n = 3.
*, p<0.05; **, p<0.01; ***, p<0.001 vs.blank.
#, p<0.05; ##, p<0.01; ###, p<0.001 vs. LPS+vehicle.

Table 11: One-way ANOVA analysis of Figure. 6B

Bonferroni's Multiple Comparison Test	Significant? p<0.05	Summary
● 12h:		
blank vs LPS	No	ns
blank vs LPS+vehicle	No	ns
blank vs LPS+psiRNA-*Dcf1*	No	ns
LPS vs LPS+vehicle	No	ns
LPS vs LPS+psiRNA-*Dcf1*	No	ns
LPS+vehicle vs LPS+psiRNA-*Dcf1*	Yes	**
● 24h:		
blank vs LPS	No	ns

blank vs LPS+vehicle	No	ns
blank vs LPS+psiRNA-*Dcf1*	No	ns
LPS vs LPS+vehicle	No	ns
LPS vs LPS+psiRNA-*Dcf1*	No	ns
LPS+vehicle vs LPS+psiRNA-*Dcf1*	No	ns
● 36h:		
blank vs LPS	No	ns
blank vs LPS+vehicle	No	ns
blank vs LPS+psiRNA-*Dcf1*	No	ns
LPS vs LPS+vehicle	No	ns
LPS vs LPS+psiRNA-*Dcf1*	No	ns
LPS+vehicle vs LPS+psiRNA-*Dcf1*	No	ns
● 48h:		
blank vs LPS	No	ns
blank vs LPS+vehicle	No	ns
blank vs LPS+psiRNA-*Dcf1*	Yes	***
LPS vs LPS+vehicle	No	ns
LPS vs LPS+psiRNA-*Dcf1*	Yes	***
LPS+vehicle vs LPS+psiRNA-*Dcf1*	Yes	###

Data are expressed as the mean ± SEM. n = 8.
*, p<0.05; **, p<0.01; ***, p<0.001 vs.blank.
#, p<0.05; ##, p<0.01; ###, p<0.001 vs. LPS+vehicle.

Table 12: One-way ANOVA analysis of Figure. 7B

Bonferroni's Multiple Comparison Test	Significant? p<0.05	Summary
blank vs LPS	No	ns
blank vs LPS+vehicle	No	ns
blank vs LPS+psiRNA-*Dcf1*	No	ns
LPS vs LPS+vehicle	No	ns
LPS vs LPS+psiRNA-*Dcf1*	No	ns
LPS+vehicle vs LPS+psiRNA-*Dcf1*	Yes	*

Data are expressed as the mean ± SEM. n = 4.
*, p<0.05; **, p<0.01; ***, p<0.001 vs.blank.
#, p<0.05; ##, p<0.01; ###, p<0.001 vs. LPS+vehicle.

Table 13: One-way ANOVA analysis of Supplement Figure. 1

**Bonferroni's Multiple Comparison Test**	**Significant? p<0.05**	**Summary**
blank vs LPS	No	ns
blank vs LPS+vehicle	No	ns
blank vs LPS+psiRNA-*Dcf1*	Yes	*
LPS vs LPS+vehicle	No	ns
LPS vs LPS+psiRNA-*Dcf1*	Yes	***
LPS+vehicle vs LPS+psiRNA-*Dcf1*	Yes	**

Data are expressed as the mean ± SEM. n = 4.
*, p<0.05; **, p<0.01; ***, p<0.001 vs. LPS+vehicle.
